# Localized eosinophilic cellulitis treated with topical ruxolitinib: A case report

**DOI:** 10.1016/j.jdcr.2024.12.020

**Published:** 2024-12-30

**Authors:** John Monroe, Anne St Pierre, Amanda Hernandez, Hannah Singer

**Affiliations:** a Norton College of Medicine, Upstate Medical University, Syracuse, New York; b Empire Dermatology, East Syracuse, New York; c ProPath, Dallas, Texas

**Keywords:** eosinophilic cellulitis, eosinophilic infiltrates, JAK inhibitors, topical ruxolitinib, Wells syndrome

## Introduction

Eosinophilic cellulitis, also known as Wells syndrome, is a rare inflammatory disorder of unknown etiology.^1^ It often presents with recurrent episodes of pruritus, erythema, and edema that can mimic bacterial cellulitis or autoimmune blistering disorders. Skin biopsy typically demonstrates an abundance of eosinophils, sometimes forming characteristic “flame figures” of eosin-staining granules around collagen.^2^ The natural course of eosinophilic cellulitis is unpredictable but can progress through acute, subacute, and chronic phases.^3^ Eosinophilic cellulitis is challenging to diagnose and treat. Standard treatment for eosinophilic cellulitis often includes systemic corticosteroids,^4^ however, relapse is common and side effects limit prolonged use.^5^ More recently, the use of systemic Janus kinase (JAK) inhibitors has been studied to treat eosinophilic cellulitis.^6^ This case describes successful management of localized eosinophilic cellulitis with topical ruxolitinib cream.

## Case report

**Fig 1:**
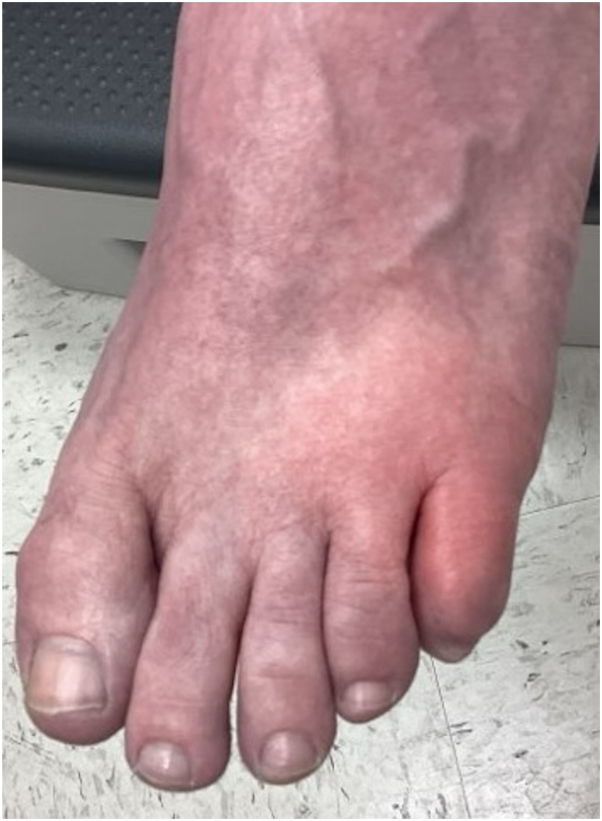
Early flare of eosinophilic cellulitis with orange-pink edema expanding from the fifth digit of the left foot.

**Fig 2:**
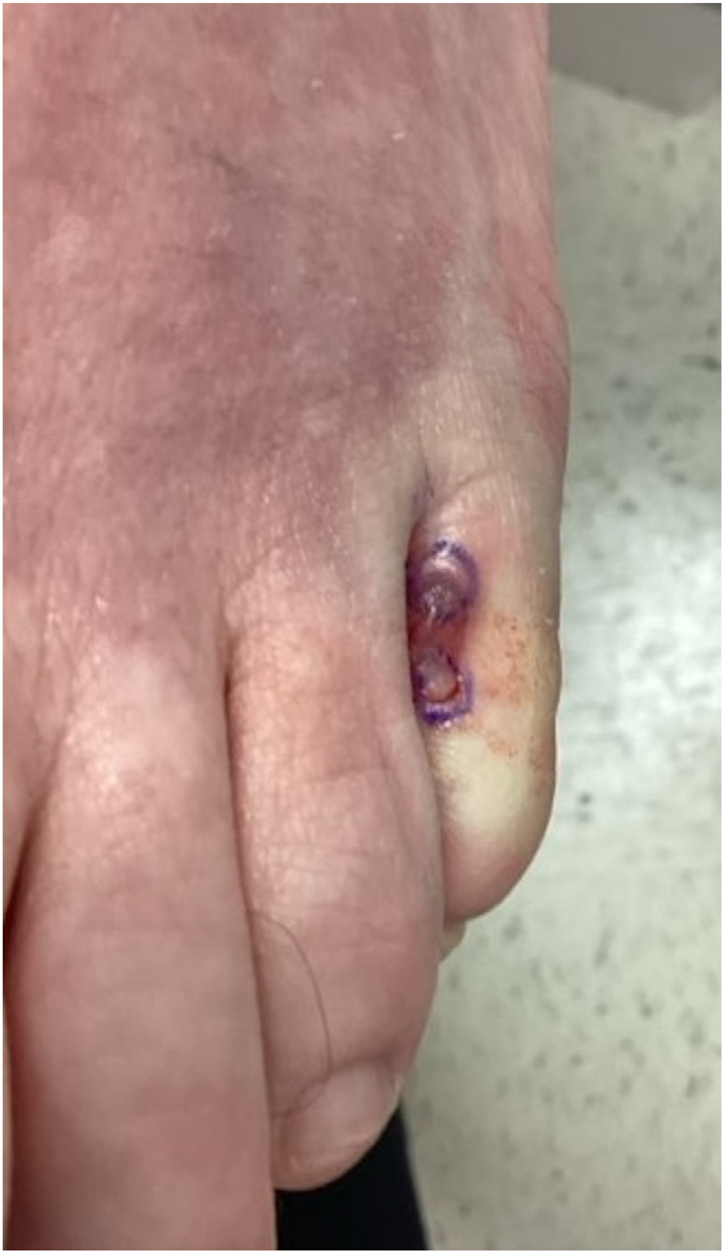
Site marked for punch biopsies at the edge of bullae, area is blanched from injection of lidocaine with epinephrine.

A 59-year-old woman presented to dermatology with a history of recurrent episodes of erythema, swelling, pain, and pruritus on her feet and lower extremities. She reported the inflammation starts as an itchy blister, then spreads with swelling to the surrounding skin. She has a past medical history of atopic dermatitis, autoimmune thyroiditis, hypertension, and colitis. Initial cutaneous examination was significant for minimal erythema with scale that was clinically consistent with tinea pedis and treated empirically with clotrimazole cream. However, several weeks later, the patient experienced an acute episode of left foot erythema, edema, and pain (Fig 1) concerning for cellulitis, for which she was admitted to the hospital for intravenous antibiotics after worsening on oral antibiotics. She improved and was discharged, but several weeks later the same area developed another erythematous, nonpurulent bulla with pruritus (Fig 2), at which point blood work, wound culture, and skin biopsies were obtained.

**Fig 3:**
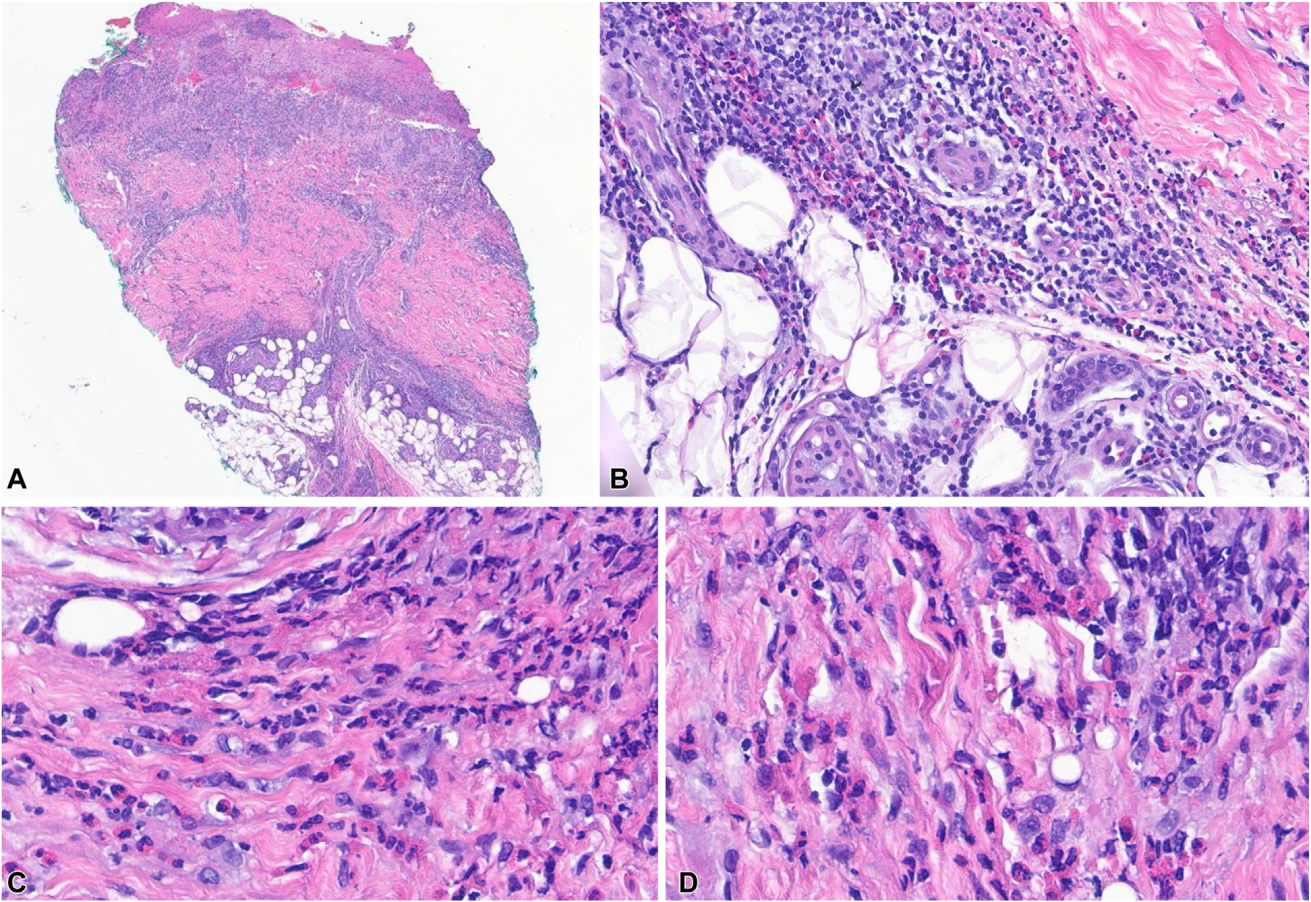
**A,** There is a brisk inflammatory infiltrate with many eosinophils involving the papillary and reticular dermis and extending into the subcutaneous adipose tissue. The roof of the blister is detached and not present in this image. **B,** The infiltrate is composed of numerous eosinophils admixed with lymphocytes and histiocytes. **C, D,** On high power there are multiple areas with eosinophils degranulating. (**A-D,** Hematoxylin-eosin stain; original magnifications: **A,** ×20; **B,** ×200; **C** and **D,** ×400.)

Laboratory studies were negative including blood cultures and Lyme serologies, and complete blood count was within normal limits. Wound culture from the interdigital toes showed normal flora, *Klebsiella oxytoca*, and *Enterococcus faecalis*, which were thought to be nonpathologic colonizers in this anatomic site. Punch biopsy of the left medial fifth toe revealed papillary and reticular perivascular dermatitis with numerous eosinophils and a subepidermal blister (Fig 3, *A*, *B*). In the reticular dermis and subcutis there were numerous degranulating eosinophils admixed with lymphocytes and histiocytes (Fig 3, *C*, *D*) No fungus was seen on periodic acid-Schiff stain. Direct immunofluorescence was negative. The histologic differential included bullous arthropod reaction, but in the clinical context the diagnosis was most consistent with eosinophilic cellulitis.

Once the diagnosis of eosinophilic cellulitis was established, treatment with topical clobetasol ointment, tacrolimus ointment, and cetirizine was trialed without significant improvement. Prednisone for severe flares was effective at symptom reduction, however, the side effects were intolerable. Based on reported literature and with the patient’s history of atopic dermatitis, dupilumab was initiated.^7^ The severity of her episodic symptoms of pruritus and swelling improved somewhat, however, the patient experienced a side effect of worsening knee arthritis and dupilumab was discontinued. Other treatment options including colchicine, cyclosporine, and dapsone were considered but the patient preferred topical treatments to minimize immune suppression and possible gastrointestinal side effects. Because clobetasol and tacrolimus ointment had not been effective, the patient was started on twice daily application of ketoconazole cream and topical ruxolitinib. Although neither hyphae nor yeast were identified on biopsy, ketoconazole cream was added in case fungal organisms were a possible trigger since these flares only occurred on her feet. Several days after starting topical ruxolitinib and ketoconazole treatment, she experienced improvement including a significant reduction in erythema, pruritus, and flare intensity without side effects. Although concomitant application of a topical antifungal agent may have contributed to the clinical response by eliminating a potential trigger, the patient discontinued use of all topicals when her symptoms improved. In follow-up, she reports when her symptoms start, she resumes use of the topical ruxolitinib only twice daily and experiences rapid relief.

## Discussion

JAK signaling interference with topical and oral small-molecule inhibitors is rapidly changing the treatment algorithms for both common and rare cutaneous inflammatory disorders. This case report describes use of a topical JAK inhibitor for eosinophilic cellulitis. Morot et al^6^ suggests that eosinophilic cellulitis is related to the activation of the JAK 1/2 signal transducer and activator of transcription proteins 5 pathways. Further, interleukin 2 is a downstream mediator of the JAK/signal transducer and activator of transcription proteins 5 pathway and activates JAK1 and JAK3 through phosphorylation, which may enhance the eosinophil response.^8^ Eosinophilic cellulitis is also associated with a variety of hematologic malignancies, malignant tumors, and eosinophilic diseases, among others. The pathogenesis of eosinophilic cellulitis is hypothesized to be due to abnormal eosinophil cell proliferation due to one of many possible triggering factors.^4^ Although the exact pathologic mechanism is unclear, ruxolitinib has proven useful for treating diseases associated with eosinophilic cellulitis, such as polycythemia vera.^9^ As ruxolitinib acts by competitively inhibiting JAK1 and JAK2 protein kinases, it may target a key inflammatory cascade in eosinophilic cellulitis.

Some oral JAK inhibitors have been associated with prothrombotic side effects in patients with cardiovascular risk factors, malignancies, immunosuppression, and other conditions.^10^ To our knowledge, ruxolitinib is the first JAK inhibitor approved by the US Food and Drug Administration as a cream formulation indicated to treat mild to moderate atopic dermatitis and vitiligo, and is far less likely to cause significant systemic side effects when used on limited surface areas.^11^ Topical ruxolitinib may be considered as a treatment option for patients with limited eosinophilic cellulitis who have not responded to topical corticosteroids, antihistamines, and who wish to avoid systemic medications.

This case report describes the use of topical ruxolitinib for treatment of recurrent localized eosinophilic cellulitis of the foot. Further investigation of topical JAK inhibitors for eosinophilic cellulitis may support its utility. Topical JAK inhibitors such as tofacitinib can be obtained through compounding pharmacies and are promising for future practice. Effective topical treatments for eosinophilic cellulitis are limited, and topical JAK inhibitors may be a safe option to trial in patients who do not respond favorably to topical corticosteroids and topical calcineurin inhibitors.

## Conflicts of interest

Dr Singer is a subinvestigator in clinical research sponsored by Loreal, Pfizer, and Dermbiont. The other authors have no conflicts of interest to declare.
